# Supplementary material for: Growth differentiation factor 15 increases in both cerebrospinal fluid and serum during pregnancy
Source: PLoS One. 2021 May 27;16(5):e0248980. doi: 10.1371/journal.pone.0248980 (PMC8158880; doi:10.1371/journal.pone.0248980)
Supplement: S1 Table — (DOCX) [file pone.0248980.s001.docx]

**S1 Table.** Pearson correlations between GDF15 and other measurements at follow-up

|  | R | *p* |
| --- | --- | --- |
| *Serum GDF15* |  |  |
| p-Glucose | -0.027 | 0.901 |
| s-Insulin | -0.089 | 0.672 |
| HOMA-IR | -0.040 | 0.853 |
| HOMA-B | -0.135 | 0.528 |
| s-Leptin | 0.180 | 0.388 |
| s-Adiponectin | -0.179 | 0.392 |
| s-Hs-CRP | -0.009 | 0.967 |
|  |  |  |
| *CSF GDF15* |  |  |
| CSF-Insulin | -0.359 | 0.172 |
| CSF-Leptin | -0.181 | 0.487 |
