# Supplementary material for: Growth differentiation factor 15 increases in both cerebrospinal fluid and serum during pregnancy
Source: PLoS One. 2021 May 27;16(5):e0248980. doi: 10.1371/journal.pone.0248980 (PMC8158880; doi:10.1371/journal.pone.0248980)
Supplement: S2 Table — (DOCX) [file pone.0248980.s002.docx]

**S2 Table.** Pearson correlations between GDF15 and self-reported dietary intake

|  | **Pregnancy** | | **Follow-up** | |
| --- | --- | --- | --- | --- |
|  | R | *p* | R | *p* |
| *Serum GDF15* |  |  |  |  |
| Energy intake | 0.295 | 0.206 | -0.014 | 0.946 |
| Carbohydrate intake | 0.322 | 0.166 | 0.192 | 0.358 |
| Protein intake | 0.387 | 0.092 | -0.220 | 0.292 |
| Fat intake | 0.201 | 0.395 | -0.123 | 0.558 |
|  |  |  |  |  |
| *CSF GDF15* |  |  |  |  |
| Energy intake | 0.071 | 0.766 | 0.040 | 0.883 |
| Carbohydrate intake | 0.078 | 0.745 | 0.165 | 0.543 |
| Protein intake | 0.097 | 0.684 | -0.032 | 0.907 |
| Fat intake | 0.045 | 0.851 | -0.058 | 0.831 |
